# Iron Deficiency Anemia in Pregnancy

**DOI:** 10.7759/cureus.28918

**Published:** 2022-09-08

**Authors:** Akshara K Raut, Keshao M Hiwale

**Affiliations:** 1 Department of Medicine and Surgery, Jawaharlal Nehru Medical College, Datta Meghe Institute of Medical Sciences, Wardha, IND; 2 Department of Pathology, Jawaharlal Nehru Medical College, Datta Meghe Institute of Medical Sciences, Wardha, IND

**Keywords:** metabolism, treatment, diagnosis, pregnancy, iron deficiency anemia

## Abstract

Through its functions in oxygen delivery, electron transport, and enzymatic activity, iron is crucial for the operation of all cells. High metabolic rate cells need more iron and are more likely to malfunction when there is an iron deficit. Since the mother's blood volume expands during pregnancy, and the fetus grows and develops, there is a sharp increase in the need for iron. Negative pregnancy outcomes, such as increased maternal sickness, low birthweight, preterm, and intrauterine growth restriction, are linked to iron deficiency. IDA, or nutritional iron deficiency anemia, affects more than two billion people worldwide and is the most prevalent disease. Based on the regression-based analysis, the prevalence of anemia in the World Health Organization's global database was calculated to be 14%. According to recent data, there are 17.4% more IDA cases among pregnant women in industrialized countries than in poor ones (up to 56% more cases). Despite the fact that oral iron supplementation is frequently used to treat IDA, not all patients benefit from oral iron therapy. This is caused by a number of things, which include the adverse effects of oral iron, resulting in low compliance and ineffectiveness. In a sizable group of patients taking oral iron preparations, the adverse effects primarily include GI symptoms. Intravenous iron was previously underutilized since it was linked to unfavorable and occasionally dangerous side effects. New type II and III iron complexes have been created recently, though, and they offer superior compliance and toleration in addition to strong efficacy and a good safety profile.

## Introduction and background

The World Health Organization (WHO) defines anemia as hemoglobin less than 11 g/dL. Iron deficiency anemia (IDA) is brought on by malnutrition, parasites, chronic illnesses, and malaria. In developing nations, anemia affects more than two-thirds of expectant mothers, with 95% of cases being caused by iron deficiency. The first postpartum week sees an iron deficiency in about 84% of women. According to the National Family Health Survey-4 (NFHS-4, 2015-2016), prenatal women in India are anemic in 45.7% of urban and 52.1% of rural areas. Globally, maternal mortality and feto-maternal morbidity are attributed to IDA directly (20%) and indirectly (50%). Hemoglobin levels rise by 0.3-1.0 g per week as a result of oral iron. Poor compliance (22-64%) brought on by gastrointestinal side effects is one of its limitations [1]. Iron stores in the mother at the time of conception and the quantity of iron absorbed throughout gestation are the two known factors that contribute to the development of iron deficiency anemia (IDA) in pregnancy. Anemia during pregnancy is a common occurrence among women in developing nations, which suggests that preexisting iron stores are frequently insufficient and that physiologic changes brought on by pregnancy are insufficient to fulfill the increasing requirements. In order to prevent the development of iron deficiency anemia, iron supplementation during pregnancy has thus become a common and normal practice. In light of the aforementioned, a review of the efficacy of therapies for iron deficiency anemia in pregnancy was undertaken. Additionally, limitations were identified and recommendations for improvement were made [2].

## Review

Iron deficiency in women

More than two billion individuals globally, particularly pregnant women, suffer from nutritional iron deficiency, the most widespread deficiency illness. Iron deficiency anemia (IDA) during pregnancy is a serious issue around the world, according to data from the World Health Organization (WHO), with prevalence rates ranging from an average of 14% of pregnant women in industrialized countries to an average of 56% (range 35-75%) in developing countries [3]. Furthermore, IDA is thought to be the only nutrient deficit that is highly frequent in both the developed and developing worlds, affecting a lot of mothers and children in both. As more than 2 billion people, or over 30% of the world's population, are iron deficient, with varying frequency, distribution, and contributing causes in different parts of the world, the number of patients with ID and IDA is staggering [4].

More women than any other ailment experience iron deficiency, which creates an epidemic public health concern. Despite numerous WHO warnings and awareness campaigns, it is typically present with modest symptoms and should be considered a chronic, slowly developing disease that is frequently ignored and neglected globally. Due to the high frequency of IDA in women, there are serious health repercussions and ensuing socioeconomic risks, such as unfavorable pregnancy outcomes, low academic performance, and reduced job capability and productivity. Numerous international nutrition conferences have addressed this topic in an effort to lessen the prevalence of iron deficiency in women of childbearing age, but without much success due to the severity and effects of iron deficiency anemia around the world, especially in women in their reproductive years. Numerous studies have been conducted on IDA's effects. Data on its impacts on patients' wellbeing, however, are still lacking [5].

Iron metabolism

Three factors - nutritional intake, iron loss, and current demand - reflect the balance of iron metabolism in healthy persons. The amount of food that has been digested and the body's capacity to absorb iron influence the nutritional iron intake. The quantity of iron absorbed mostly depends on whether the digestive system is diseased or whether a comorbidity (such as chronic inflammatory disorders) may trigger the development of iron regulatory proteins and a peptide called hepcidin, which ultimately inhibits iron absorption [6]. In humans, erythrocytes are destroyed by macrophages of the reticuloendothelial system, including the spleen, which results in a renewed internal iron supply. Recent research has demonstrated how intestinal and hepatic proteins are used by the human body to up- and down-regulate iron absorption in response to altering iron status [7-8].

Iron Metabolism in Pregnancy

Fetal hepcidin regulates the placental transport of iron from maternal plasma to the fetal circulation throughout pregnancy. The rate at which iron enters the plasma increases when hepcidin levels are low. Iron is trapped in enterocytes, macrophages, and hepatocytes when hepcidin levels are high [9]. Ferroportin is internalized when hepcidin levels are high. The minimal daily requirement for exogenous iron still ranges from 1 to 8 mg. To balance the increasing need for iron, especially with the physiological needs during development, pregnancy, and lactation, extra exogenous iron is however necessary. This markedly increased need for iron is necessary to support the mother's blood volume as well as the growth of the fetus and placenta. Additionally, iron loss occurs in pregnant women both before and after birth [10-11]. About 1000 mg of iron are lost overall during pregnancy and lactation. As a result, pregnant women should consume 27 mg of iron daily rather than the 8 mg that adults who are not pregnant should consume. A daily food intake of 10 mg is necessary for lactation [12].

Laboratory markers for iron status

Definition of Anemia in IDA in Pregnant and Nonpregnant Women

According to various clinical practice recommendations, anemia in pregnancy is commonly described as Hb 11 g/dl or 11.5 g/dl, with a slight variation depending on the trimester of pregnancy. However, if a hemoglobin level is below 100 g/L at any point in pregnancy, anemia should be investigated and treated due to the possibility of catastrophic effects for both the mother and her unborn child, including a higher risk of intrauterine growth retardation and premature birth. As a temporary measure, anemia in women of reproductive age is defined as Hb 120 g/L or, in some studies, 115 g/L [13-14].

Definition of Iron Deficiency (ID)

Severe iron deficit is defined as a serum ferritin level below 20-30 g/L and mild-moderate iron shortage as a level below 70-100 g/L. The ferritin level is used as a stand-in marker for ID. A concurrent test for inflammatory markers is advised in cases of anemia with high ferritin to rule out reactive causes because serum ferritin is an acute phase reactant and may be elevated in cases of inflammation or infection. If the ferritin level is greater than 100 g/L, ID is probably absent [15]. Despite being the most reliable indicator of iron insufficiency, a study of bone marrow iron reserves is still impractical and intrusive for the majority of patients. A method for precise IDA diagnosis is the measurement of serum ferritin and soluble transferrin receptors. Transferrin receptor testing, however, is not a procedure that can be consistently and accurately duplicated in the majority of laboratories around the world [16].

While ferritin estimation is a simple automated test that can be carried out in the majority of laboratories around the world, its application is restricted in cases of inflammation or infection because it is thought to be influenced by acute phase reactions, which reduces its value in the clinical interpretation of the test results. Serum iron, transferrin, total iron-binding capacity (TIBC), transferrin saturation, and ferritin are the generally used laboratory assays that can be used to detect an individual's iron status [17]. Human plasma contains soluble TfR (sTfR), which is thought to be a shortened version of the tissue receptor that exists as a transferrin-receptor complex and hence reflects tissue iron deficit. Hepcidin, which is largely produced by hepatocytes and secreted into the blood circulation, is another protein that is essential for iron metabolism. Hepcidin is a tiny molecule with a 25-amino acid peptide structure that is secreted by the kidneys and can therefore be found and tested in urine [14, 15]. Furthermore, the quick excretion of hepcidin indicates that the regulation is initiated at the level of the production sites. Hepcidin is a circulating protein in the ferroportin plasma that reacts to a number of stimuli to control iron storage and serum iron levels [18-19].

Current Strategy to Assess Iron Deficiency During Pregnancy

An excellent screening technique for IDA is a full blood count and mean corpuscular volume (MCV) value allowing the identification of microcytic anemia. However, iron studies, particularly ferritin level, remain the surrogate marker for IDA in regions of the world where haemoglobinopathies are common and they may be associated with microcytosis [20]. Iron deficiency can be categorized as severe ID when the ferritin level is below 30 g/L or mild-moderate ID if the ferritin level is between 100 g/L and 30 g/L (there is a wide normal range between 20 and 464 and is laboratory and method specific). A reactive common cause, such as infection, should be ruled out in cases of increased ferritin >100 g/L with concurrent anemia, and other causes of anemia should then be looked into. The diagnosis of IDA can be confirmed by further iron-related assays, such as serum iron, iron-binding capacity, and transferrin saturation [21].

Intravenous Versus Oral Iron Therapy in Pregnancy

Based on Hb levels, intravenous iron, including iron sucrose, was used in randomized controlled trials with improved efficacy compared to oral iron alone or in combination with intravenous iron [22]. An increased incidence of thrombosis has been linked to a single IV iron-sucrose dosage. Contrarily, intravenous iron sucrose given in six tiny doses over the course of three weeks did not cause infusion-associated thrombosis, and it was well tolerated when given to 45 pregnant women in five daily doses. There was no discernible change in Hb levels between intravenous iron sucrose and oral iron sulfate at any time tested at days 8, 15, 21, and 30 and at delivery in a study using intravenous iron sucrose. However, when Hb levels were assessed at two and four weeks following IV iron administration and at delivery in a different experiment using six tiny doses of iron sucrose, there was a substantial difference in favor of the intravenous iron sucrose group. However, both studies' administration of IV iron sucrose came at the expense of much greater patient effort to visit the hospital for six infusions quickly and the increased demands on hospital resources [22-23].

According to the data, 79% of women who got oral iron treatment had lower-than-normal ferritin levels at delivery compared to 4.5% of women who had IV iron treatment (P 0.001). In the oral iron group, 29% of pregnant women had Hb levels below 116 g/L, compared to 16% in the IV iron group (P = 0.04). Despite the high incidence and burden of the disease associated with IDA, a thorough meta-analysis revealed a dearth of high-quality trials investigating the clinical maternal and neonatal effects of iron therapy in women with IDA. Only one prospective randomized trial comparing IV iron to oral iron for treating IDA during pregnancy throughout this time period met the strict independent reviewer quality requirements [24].

Side Effects of IV Iron

Intravenous iron was only sometimes used in the past because of its unfavorable and occasionally serious adverse effects. However, new type II and type III iron complexes have been created recently that are more tolerable and can be employed for quick replenishment of iron reserves. Intravenous iron continues to be underutilized due to prior worries about the acceptability of earlier intravenous iron preparations, despite mounting evidence of the safety of the newer preparations in both pregnant and general populations [25].

A review of 481 patients of both sexes who had iron dextran infusions found that roughly 25% of patients experienced modest, self-limiting adverse effects. However, roughly 2% of people had really severe allergic reactions, and 0.6% of those were deemed to be anaphylactic. The majority of these responses happened right away during the test dose's infusion [16]. With only 3.3% allergic reactions reported per million doses per year with iron gluconate, it is thought to have a lower reaction rate, hence a test dosage is not advised. The infusion of iron gluconate was not associated with any responses that could have been fatal. On the other hand, iron dextran was associated with 196 allergic/anaphylactic responses and 31 fatalities. The use of iron dextran in pregnancy has been constrained due to the high frequency of adverse reactions, including significant adverse events. Although iron gluconate administration is thought to be safe, it is nonetheless impracticable in practice due to the need for several infusions, which has a significant impact on both patient compliance and the frequently constrained resources of the healthcare system [26].

Avoiding Blood Transfusion

The conventional effective method for treating anemia in patients with severe IDA has been a blood transfusion, particularly in cases when patients have not responded to dietary iron therapy or when a rapid anemia correction is clinically necessary. The avoidance of blood transfusions during pregnancy is not well understood, however, a recent experiment comparing the treatment of IDA with oral versus IV iron in pregnancy found that neither treatment arm's participants received blood transfusions to manage anemia during pregnancy. However, in the postpartum phase, two individuals (0.9%) in the oral iron group required blood transfusions. To attain the best therapeutic results, safe, effective, larger, and less frequent doses must be used in a variety of clinical circumstances. These techniques' main objectives include lowering total costs, providing relief to overburdened health systems, enhancing patient convenience, enhancing compliance, maintaining venous access, and minimizing blood transfusions [27].

Discussion

**Figure 1:**
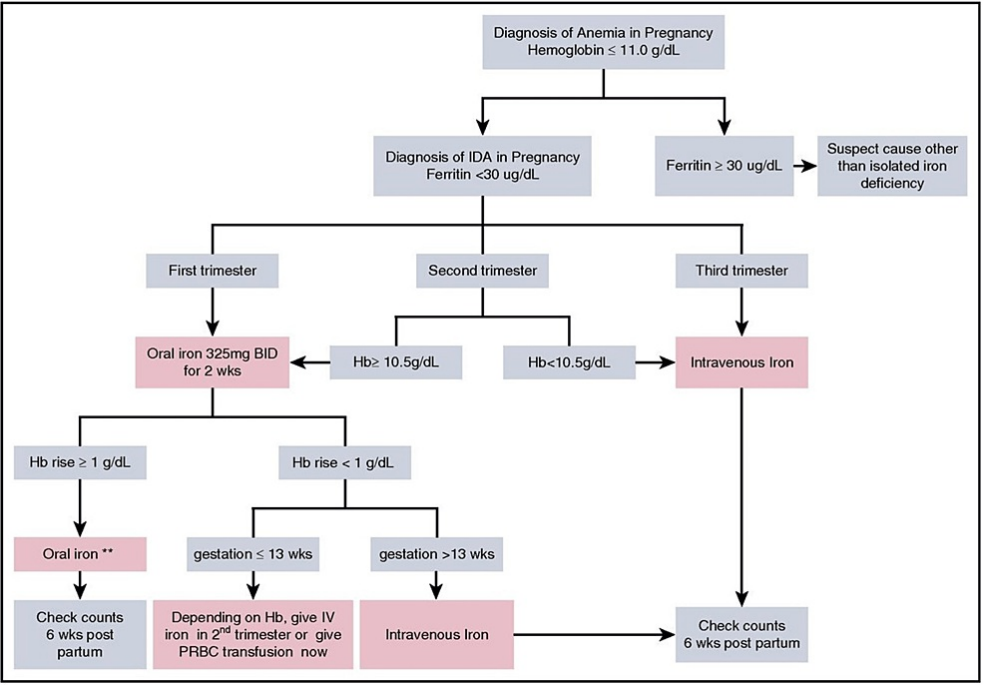
Suggested approach to diagnosis and management of iron-deficiency anemia in pregnancy

The WHO has identified IDA as the most crippling nutritional deficit in the world in the twenty-first century, highlighting that women are disproportionately at risk. Such a situation can have disastrous effects on entire populations and major repercussions if it is ignored and improperly treated. Therefore, in some clinical circumstances, intravenous iron therapy should be thought of as a quick, efficient, and secure treatment alternative. Based on various prospective randomized trials, an approach for treating iron deficiency anemia in pregnancy and the postpartum period is suggested (Figure 1). Intravenous iron is being used more frequently to prevent or lessen the need for blood transfusions and to effectively replenish iron levels quickly. The recently created intravenous iron formulations, which are regarded as a milestone in the therapy of IDA [28], should be taken into consideration while considering treatment options for IDA.

Overall, communities in the developing world are the most at risk, particularly the poorest and least educated groups who are disproportionately impacted by iron deficiency and thus stand to benefit the most from the eradication of IDA. In addition, recognising the scope and severity of the IDA problem during pregnancy as well as in the non-pregnant female population will assist health professionals in identifying the most effective diagnostic and therapeutic approaches, which are essential in overcoming such a severe health issue. A worldwide approach to the health and economic aspects of IDA should be taken into consideration, along with a consensus guideline developed by international specialists in managing IDA in women and the general population that incorporates novel intravenous iron therapy [28-29].

## Conclusions

It is worthwhile to take into account a global, all-encompassing IDA management algorithm that provides several evidence-based treatment alternatives and attends to regional issues. However, resource shortages are a common problem in poor nations where IDA is widespread. Therefore, it is essential to design a workable programme with the intention of successfully utilising the local resources available. The key to the effectiveness and sustainability of such a programme may lie in making the treatment of IDA a top priority and raising community awareness of such a persistent, grave condition. Without a doubt, the successful eradication of IDA will have a significant positive impact on productivity and community health, as well as result in significant health savings in both developing and wealthy countries.
